# The Comparative Diagnostic Features of Canine and Human Lymphoma

**DOI:** 10.3390/vetsci3020011

**Published:** 2016-06-09

**Authors:** Davis M. Seelig, Anne C. Avery, E. J. Ehrhart, Michael A. Linden

**Affiliations:** 1 Department of Veterinary Clinical Sciences, College of Veterinary Medicine, University of Minnesota, St. Paul, MN 55455, USA; 2 Department of Microbiology, Immunology, and Pathology and the Flint Animal Cancer Center, College of Veterinary Medicine and Biomedical Sciences, Colorado State University, Fort Collins, CO 80523, USA; anne.avery@colostate.edu (A.C.A.); ej.ehrhart@colostate.edu (E.J.E.); 3 Department of Laboratory Medicine and Pathology, University of Minnesota, Minneapolis, MN 55455, USA; linde013@umn.edu

**Keywords:** lymphoma, comparative oncology, canine, non-Hodgkin lymphoma, comparative pathology

## Abstract

The non-Hodgkin lymphomas (NHLs) are a heterogeneous family of lymphoid malignancies that are among the most common neoplasms of both dogs and humans. Owing to shared molecular, signaling, incidence, and pathologic features, there is a strong framework supporting the utilization of canine lymphoma as a comparative, large animal model of human NHL. In alignment with the biologic similarities, the current approach towards the diagnosis and classification of canine lymphoma is based upon the human World Health Organization guidelines. While this approach has contributed to an increasing appreciation of the potential biological scope of canine lymphoma, it has also become apparent that the most appropriate diagnostic philosophy must be multimodal, namely by requiring knowledge of microscopic, immunophenotypic, and clinical features before establishing a final disease diagnosis. This review seeks to illustrate the comparative similarities and differences in the diagnosis of canine lymphoma through the presentation of the microscopic and immunophenotypic features of its most common forms.

## 1. Introduction

**Figure 1:**
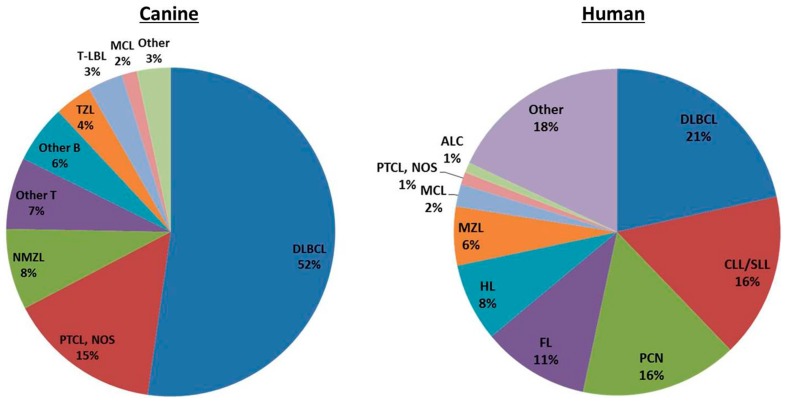
Incidence of canine (**A**) and human (**B**) lymphoma. Canine data reflect summary data from three publications whereas human data originate from one. DLBCL; diffuse large B-cell lymphoma, PTCL, NOS; peripheral T-cell lymphoma, not otherwise specified, NMZL; nodal marginal zone lymphoma, TZL; T-zone lymphoma, T-LBL; T-cell lymphoblastic lymphoma, MCL; mantle cell lymphoma, CLL/SLL; chronic lymphocytic leukemia/small lymphocytic lymphoma, PCN; plasma cell neoplasia, FL; follicular lymphoma, HL; Hodgkin’s lymphoma, and ALC; anaplastic large cell lymphoma.

The non-Hodgkin lymphomas (NHL) are a heterogeneous family of related, yet very distinct, lymphoid malignancies that are among the most common neoplasms in both human and dog. In humans, NHL is the 7th most common cancer type, representing 4.3% of all new cancer cases with an annual incidence of 19.7 per 100,000 [1]. Although the true incidence of canine lymphoma is somewhat uncertain as most dogs with the disease are not reported, most studies agree that lymphoma is the most common hematopoietic neoplasm of the dog and among the three most common canine neoplasms [2,3,4,5,6]. The largest, and most widely cited studies, report that lymphoma represents approximately 6% of all canine malignancies and 90% of all hematopoietic neoplasia with an annual incidence up to 24 cases per 100,000 dogs [4,5,7]. In addition to similar incidence rates, there is compelling evidence that the incidence of lymphoma is increasing in both species. The most recent data from a pet insurance study reports that the age-adjusted incidence of canine lymphoma has increased to 107 cases per 100,000 dogs at risk [8] whereas the incidence of human NHL has increased in the past four decades in both the USA and other countries, including a nearly doubling of the incidence rate between 1970 and 1990 [9]. In addition to their shared incidence, there is a robust and ever-expanding literature describing the shared biologic, genetic, and, in accordance with this review, their shared pathology. In both species diffuse large B-cell lymphoma (DLBCL) is the most common subtype (Figure 1), but this is where the similarities end [10,11,12,13].

In the dog, the next most common subtypes, namely peripheral T-cell lymphoma, not otherwise specified (PTCL, NOS), nodal marginal zone lymphoma (NMZL), and T-zone lymphoma (TZL), the latter of which is considered a morphologic variant of PTCL, NOS in humans, are much less common in humans. In light of the rapidly evolving field of canine diagnostic hematopathology, the expanding access to advanced immunophenotyping options, and an increasing utilization of a standardized diagnostic algorithm (see below), it is likely that many of the published large canine studies that form the basis of current incidence data underestimate certain forms of lymphoma, including those with novel histologic architectures (e.g., nodular forms of lymphoma).

## 2. Classification of Canine and Human Lymphoma

According to the most recent World Health Organization (WHO) diagnostic guidelines, there are over 60 forms of human NHL. While it is beyond the scope of this review to summarize the evolution of human NHL classification, the inextricable link between diagnosis and classification and recent work validating a canine version of these guidelines necessitates a brief summary of its philosophical underpinnings. The process of lymphoma classification seeks to segregate individual disease subtypes according to reproducible and clinically relevant guidelines that facilitate efficient communication and enable relevant treatment and research. According to the WHO, lymphoma subtypes are defined according to their cytomorphology, immunophenotype, genetic, molecular, and clinical features [14]. The driving philosophies of this system are two-fold, namely: (1) that neoplastic lymphocytes are malignant counterparts of benign cells at varying stages of differentiation; and (2) that morphologically-indistinguishable neoplastic cells could be characterized as different lineages according to their genetic, molecular, and immunophenotypic characteristics. Although its scope was limited by a lack of species-specific genetic and/or immunophenotypic information, recent work has confirmed the applicability of the 2008 WHO guidelines to canine lymphoma [15]. Using a canine-adapted WHO system, a series of 18 international veterinary pathologists were able to obtain an overall accuracy of 83% with inter-observer variability of 65% when provided glass slides and immunophenotype information for 300 cases of canine lymphoma. This accuracy was increased to 87% when the case series was limited to the six most common forms of disease [15].

Although the clinical implications of classification has been recognized for many years in human hematopathology, the increasing recognition of distinct biological behavioral differences amongst canine lymphoma subtypes is more recent. A number of reports have identified distinct subtypes of canine lymphoma with a slow rate of biologic progression, so-called “indolent” lymphomas. These include marginal zone lymphoma, mantle cell lymphoma, and T-zone lymphoma. In contrast to subtype agnostic survival times of 10–12 months for canine B-cell lymphoma and 6 months for canine T-cell lymphoma, subtype specific survival times of 21 and 33 months for indolent forms of canine B-cell and T-cell lymphoma, respectively have been reported [16].

### 2.1. Basic Histologic Approach

The current standard for the diagnosis and classification of human lymphoma, which also serves as the basis for the current canine recommendations, is the 2008 REAL-WHO guidelines [17,18]. On histologic samples, these guidelines emphasize three features, namely: (1) tumor architecture; (2) tumor cell morphology (*i.e.*, size, shape, and nuclear/cytoplasmic features); and (3) mitotic activity. These features, in concert with knowledge of immunophenotype, clinical disease burden, and, in the case of human NHL, genetic features, are used to establish a final disease diagnosis. According to the current guidelines, there nearly 50 discrete subtypes of human NHL (Table 1). Using the human WHO as a template, 20 canine diseases have been described (Table 1).

In practice, application of the WHO histologic algorithm requires 3–5 µm thick, hematoxylin and eosin (HE) stained tissue sections. It is important to note that the histologic criteria used to diagnose and classify canine lymphoma are entirely based upon the human WHO classification system and, as such, a high degree of morphologic overlap between human and canine variants of the same subtype is to be anticipated. Tissue sections from wholly excised lymph nodes are considered to be the gold-standard for the diagnosis and classification of lymphoma [18,19]. The diagnostic utility of non-excisional lymph node biopsy samples has yet to be studied in veterinary medicine.

**Figure 2:**
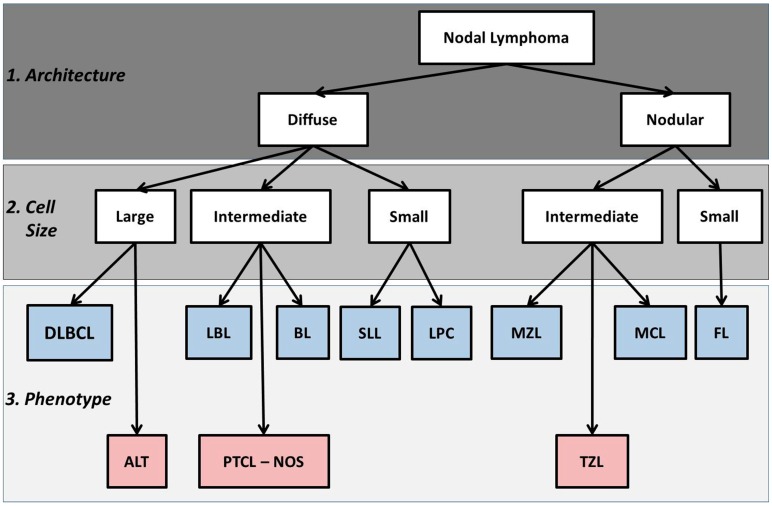
The histologic approach towards the classification of canine nodal lymphoma. Using excisional lymph node sections, lymphoma is initially divided into diffuse (effacing) or nodular (non-effacing) forms of the disease. Next, using a red blood cell or a small lymphocyte as a guideline, the neoplastic population is divided into large, small, and intermediate forms of the disease. Finally, using knowledge of additional cellular and nuclear features, including mitotic rate, and immunophenotype, a final diagnosis is established. DLBCL; diffuse large B-cell lymphoma, PTCL, NOS; peripheral T-cell lymphoma, not otherwise specified, NMZL; nodal marginal zone lymphoma, TZL; T-zone lymphoma, T-LBL; T-cell lymphoblastic lymphoma, MCL; mantle cell lymphoma, CLL/SLL; chronic lymphocytic leukemia/small lymphocytic lymphoma, PCN; plasma cell neoplasia, FL; follicular lymphoma, HL; Hodgkins lymphoma, and ALC; anaplastic large cell lymphoma.

Using the 2008 WHO guidelines as a template, canine nodal lymphoma is initially divided according to tumor architecture as either a “diffuse” or “nodular/follicular” disease (Figure 2). In the case of the former, all or nearly all of the lymph node architecture is effaced by the neoplastic infiltrate whereas in the latter, the neoplastic infiltrate is organized in fashion that either mimics, or spares some portion, of lymph node tissue. Cytologically, tumor cells are classified as either small, intermediate, or large. Neoplastic cell size is determined based upon nuclear size relative to a known standard, traditionally either red blood cell (RBC) or a small lymphocyte. In human hematopathology, the gold standard for assessing cell size is an endothelial cell nucleus, although a small lymphocyte may be used. Based upon this comparison, neoplastic cells are classified as small (nuclei approximately 1–1.25 × the diameter of a red blood cell or smaller than an endothelial cell nucleus), intermediate (nuclei approximately 1.25–2 times the diameter of a red blood cell or equal to the size of an endothelial cell nucleus) or large (nuclei ≥ 2.0 × the diameter of a red blood cell or larger than an endothelial cell nucleus). Finally, using additional cytoplasmic (volume and staining intensity) and nuclear (shape, chromatin pattern, and nucleolar features) features in concert with knowledge of immunophenotype, a final histologic diagnosis can be established (Figure 2).

Knowledge of neoplastic cell immunophenotype is an absolute requirement for the complete diagnosis and classification of lymphoma in either species. A number of tools, including flow cytometry, immunocytochemistry, and lymphoid clonality assays, can provide immunophenotype, but Immunohistochemistry (IHC) is considered the gold standard [20]. The importance of IHC in the characterization of canine lymphoma was illustrated by Flood-Knapik *et al*. which reported a reclassification of 20% of indolent lymphomas cases with the addition of IHC data [16].

### 2.2. FNAC in the Diagnosis of Lymphoma

In light of their shared pathology, the philosophical approach towards the diagnosis and classification of lymphoma in both man and dog is similar. Although there are technologic constraints and economic realities that do not allow the direct duplication of human NHL diagnostic algorithms to the dog, both human and veterinary pathologists generally adopt a stepwise approach. When faced with an enlarged peripheral lymph node, the chief objective of both is to refine the provisional list of differentials which includes reactive hyperplasia, lymphadenitis, lymphoma, and metastatic disease. Fine needle aspiration cytology (FNAC) may be suited for these initial investigations owing to its simplicity, low-cost, safety, and minimally-invasive sampling.

As the vast majority (>80%) of the cases of canine nodal lymphomas present with diffuse effacement of one or more peripheral lymph nodes by large neoplastic cells, fine needle aspiration cytology (FNAC) is a widely accepted as a highly sensitive and specific first-line lymphoma diagnostic. In humans, FNAC has a >90% sensitivity and specificity in differentiating a malignant process from reactive hyperplasia [21,22]. In both species, the results of the FNAC are often used to trigger a number of second tier studies, including flow cytometry, immunocytochemistry, biopsy with immunohistochemistry, clonality assays, and cytogenetic analysis [17]. While FNAC has numerous advantages, it is generally unsuitable as a solitary diagnostic for certain subtypes of lymphoma, including nodular and small-cell variants [23,24]. In general, while FNAC plays an important role in the management of human patients, a tissue-based diagnosis by needle core or excisional biopsy (with immunophenotyping), is essentially required prior to initiating (chemo) therapy.

### 2.3. Flow Cytometry in the Diagnosis and Classification of Lymphoproliferative Disorders

When used in concert with FNAC, flow cytometry immunophenotyping offers many advantages. Namely, it requires only a small volume of sample, it can evaluate multiple antigens on one cell, it can give quantitative results, and it can detect rare and phenotypically aberranT-cells. As an instrument of diagnostic hematopathology, flow cytometry (FC) chiefly detects and subclassifies lymphoid neoplasms through the identification of tumor-specific immunophenotypic signatures, including atypical light scatter (chiefly size) and antigen expression profiles. Atypical antigen expression is reflected by either monoclonal light chain expression (in the case of human B-cell neoplasms) or in the detection of cells with an antigen profile incongruous with normal lymphoid cells.

Monoclonal, or restricted light chain expression, is the most useful surrogate marker of B-cell clonality in humans and, in practice, is generally defined by kappa:lambda ratios of >10 or lambda:kappa ratios of >5 [25]. In contrast, given that both neoplastic and reactive B-cell populations are dominated by lambda-expressing cells, a similar approach is not feasible in dogs [26]. As such, in the evaluation of canine B-cell lymphomas, FC is chiefly used to provide adjunctive immunophenotyping and prognostic information, rather than act as a primary diagnostic tool [27]. Although a similar surrogate marker of monoclonality does not exist for T-cells, immunophenotypic aberrancy, as defined by abnormal increases or decreases (including complete loss of surface antigen expression) in normal T-cell antigens or unexpected co-expression profiles, is often used to diagnose T-cell neoplasia [28]. Using subtype-specific antigen expression profiles (Table 2), the combination of FNAC and FC has a sensitivity and specificity of 97% and 94%, respectively for the diagnosis and classification of human NHL in a series of 72 cases with histologic follow-up [28].

In canine hematopathology, although the diagnostic philosophy with respect to FC is similar, the limited species-specific reagents and the novel biological features of canine lymphoma, require an alternate approach. In contrast to human hematopathology, in which FC panels using 15–25+ antibodies (cocktailed in more than one tube) are recommended, most canine FC laboratories use panels with 10–12 antibodies [24,29]. Moreover, as the vast majority of both normal and malignant lymphoid populations express the lambda variant, light chain restriction is not a practical surrogate marker of clonality in canine lymphoproliferative diseases [26,30]. While no study has yet examined the performance of FC as a primary diagnostic tool for canine lymphoma, a number of recent studies have confirmed its utility as a tool for advanced classification of certain forms of canine lymphoma, for lineage assignment, and for the provision of prognostic information.

The sections that follow will, using an evidence-based approach, review the diagnostic approach for the most common forms of canine lymphoma. Incidence data for specific subtypes of canine lymphoma classified according to the updated WHO algorithm stems from three studies, which comprise a total of 1675 cases [11,12,13]. Using this data, this comparative diagnostic review will emphasize the most common forms of canine nodal lymphoma, namely: (1) Precursor disease; (2) DLBCL; (3) Marginal Zone Lymphoma; (4) Mantle Cell Lymphoma; (5) Peripheral T-cell Lymphoma NOS; and (6) T-zone lymphoma.

## 3. Precursor Neoplasms

Lymphoblastic lymphoma (LBL) and acute lymphoblastic leukemias (ALL) are precursor cell neoplasms derived from immature lymphoid cells of either B (B-LBL/ALL) or T-cell (T-LBL/ALL) lineage. They are believed to arise from lineage-committed precursors in either the bone marrow (B-LBL/ALL) or thymus (T-LBL/ALL). The distinction between LBL and ALL of either phenotype is based upon the tumor tissue burden rather than any unique cellular features. Namely, if the disease is dominated by blood or bone marrow involvement (*i.e.*, >20%–25% neoplastic cells), the diagnosis is ALL. Alternately, if tumor burden predominantly involves non-blood/non-marrow tissue compartments the diagnosis is LBL. Practically, the distinction between ALL/LBL is thought to be somewhat arbitrary as the biologic behavior and prognosis of LBLs and ALLs of a particular phenotype are considered to be equivalent.

The precursor neoplasms comprise approximately 2% of all human NHL and, in total, the B-cell form of the diseases predominates over the T-cell form. However, when segregated into LBL and ALL types, the incidence of the B- and T-cell forms of the disease differs [31]. With hALL, the B-cell form of the disease (hB-ALL) predominates, representing 75%–80% of all acute leukemias, whereas in contrast, hLBL is far more often a T-cell disease, with an approximate ratio of 9:1 [32]. Although they are overall uncommon neoplasms, the precursor diseases are reported to be slightly more frequent in the dog than in humans, with LBL representing 3%–9% of canine lymphomas [11,12,13]. Similar to humans, canine T-LBL (64%–100%) is reported to be much more common than canine B-LBL (0%–34%), however it is important to note that the largest effort to classify canine lymphoma using human criteria, precursor B-cell lymphomas were not present in the 600 cases studied [11,12,13]. One report suggests that cB-ALL is much more common than cT-ALL [33].

**Figure 3:**
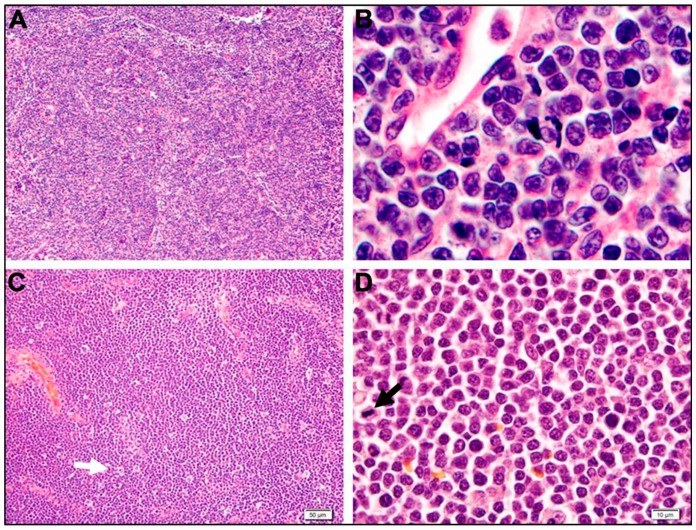
The histologic features of human and canine precursor disease (LBL/ALL). In both species (**A**, **B**, human and **C**, **D**, canine) the architecture is diffuse (**A** and **C**). Morphologically, the neoplastic cells in both species are small-to-intermediate in size with round to convoluted nuclei containing dispersed to clumped chromatin and faint to indistinct nucleoli. A starry sky appearance (white arrow; (**C**) and high mitotic rate (black arrow); (**D**) may be seen in both.

The diagnostic approach to the precursor diseases in both species is similar. In both human and canine variants, LBL is a diffuse disease comprised of a uniform population of neoplastic cells (referred to as lymphoblasts) that occasionally infiltrate the perinodal tissue (Figure 3) [34]. The cells are small-to-intermediate in size with round to convoluted nuclei containing variable (dispersed to condensed) chromatin and faint to indistinct nucleoli. Cytoplasmic volume is scant and, often, LBL has a high mitotic rate and a starry sky appearance [11,12,13,34,35,36]. Similarly, the diagnosis of either hALL or cALL necessitates the identification of >20%–30% blasts in blood or bone marrow [37,38]. In both species, the two forms of precursor disease (*i.e.*, LBL *vs.* ALL) and the two phenotypes (*i.e.*, T *vs.* B-cell) cannot be distinguished using light microscopy.

Immunophenotypically, detection of the stem cell antigen CD34 and terminal deoxynucleotidyl transferase (TdT) are hallmarks in the diagnosis of hALL/LBL [34,36]. Human B-ALL/LBL is nearly always positive for B-cell markers CD10, CD19, cytoplasmic CD79a, and CD22 with dim to absent CD45 expression, whereas T-ALL/LBL consistently express T-cell markers cytoplasmic CD3, CD5, and CD4 and CD8 [32,36]. The expression of TdT and the lack of surface IG are useful in distinguishing precursor B-cell disease from mature B-cell neoplasms, whereas the expression of TdT, CD34, and the presence of cytoplasmic CD3 without surface CD3 can be used to distinguish mature T-cell neoplasms from precursor T-cell disease [36]. The expression of CD45 in contrast, the immunophenotypic profile of the canine precursor diseases, owing to their rarity and the lack of a standardized diagnostic approach is murkier.

According to published reports, the canine precursor diseases, like their human counterparts, express either B or T-cell antigens (e.g., CD79a, CD21, CD3, and/or CD5) [33,38,39,40,41,42,43,44], but in contrast, demonstrate varied CD34 expression (*i.e.*, rare in cLBL, but common in cALL) [11,12,13,43,45]. To date, immunostaining for TdT has not proven useful in the diagnosis of canine precursor disease, although it is unclear of these negative findings reflect true biologic differences between the canine and human forms of the disease or if they represent antibody shortcomings [46]. However, it is important to emphasize that these reports utilize highly variable diagnostic techniques. For example, most reports of cLBL utilize archived, formalin-fixed, paraffin-embedded tissues, which are unsuitable for canine anti-CD34 IHC, whereas cALL studies primarily use flow cytometry, where detection of CD34 is more robust. In practice, most canine precursor neoplasms (predominantly acute leukemias) are phenotyped by flow cytometry rather than histology. While it is generally accepted that CD34 expression is diagnostic for precursor leukemia, there are no consensus criteria for differentiating true precursor leukemias from other forms of lymphoproliferative disease when CD34 expression is lacking [47]. Moreover, there is limited understanding as to the role of lineage-specific markers for the immunophenotyping of canine precursor disease. For example, although CD21 expression as detected by flow cytometry is variably used in the literature to diagnose cB-ALL, in other species CD21 is not expressed until B-cell reach maturity, and is not expressed on human B-ALL/LBL [48]. And, although CD79a is more commonly used to ascribe a B-cell lineage to human precursor disease, and may be useful in cALL [33], it has not yet been established that CD79a is specific for B-cell ALL/LBL in dogs [49].

## 4. T-Cell Neoplasms

As a percentage of all NHL, the mature T-cell lymphomas are less common than their B-cell counterparts in both species, although they are much more common in the dog than human representing 30%–40% and 6%–10% of all lymphomas by species, respectively [10,11,13,50]. In the dog, the most common forms appear to be T-zone lymphoma and PTC-NOS whereas in humans, the most common forms are PTCL-NOS and anaplastic large cell lymphoma [10].

### 4.1. Peripheral T-Cell, Not Otherwise Specified (PTCL, NOS)

While designated as a specific subtype of PTCL in the current WHO classification, PTCL, NOS is a heterogeneous group of nodal and extranodal mature T-cell neoplasms that demonstrate features which set them apart from other forms of PTCL. In both species, the category of PTC, NOS is a “wastebasket” or “catchall” category. Based upon their morphological, genetic, and immunophenotypic heterogeneity, it is accepted that PTCL, NOS contains many different unique entities, however no reliable criteria have been established that allow for consistent identification of any one specific entity. In both human and canine, peripheral T-cell lymphoma not-otherwise specified (PTCL, NOS) is the most common T-cell lymphoproliferative disease, representing approximately 30% and 39%–65% of all human and canine cases, respectively [11,13,51]. However, as a percentage of all forms of lymphoma, PTCL, NOS is uncommon in humans, representing less than 1% of all NHL, but comparatively much more common in dogs, at 15% of all lymphomas.

The cellular origins of both human PTCL, NOS (hPTCL, NOS) and cPTCL, NOS are likely heterogeneous, but are presumed to be derived from post-thymic T-cells in various stages of transformation [51]. Gene expression profiling (GEP) studies suggest that hPTCL, NOS can be subdivided into at least three cell-of-origin subgroups, namely cytotoxic, helper, and follicular helper PTCL, NOS [52,53]. Initial works by the International Peripheral T-cell Lymphoma Project suggests that molecular subtyping may have clinical implications as the cytotoxic tumors appear to behave significantly worse [54]. Clinically, patients with hPTCL, NOS most often present with disseminated disease characterized by generalized lymphadenopathy and extranodal involvement, with skin, GI tract, and bone marrow being the most commonly affected sites [17,55,56]. Similarly, canine PTCL, NOS (cPTCL, NOS) has a varied pattern of tissue involvement, including nodal and extranodal (subcutaneous, hepatosplenic, mediastinal, and intestinal) manifestations [39].

**Figure 4:**
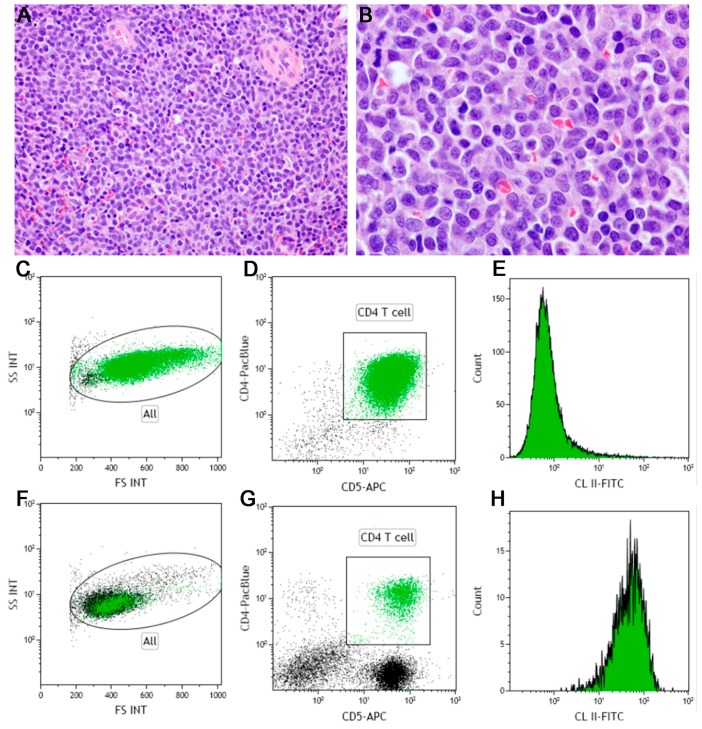
The histologic and flow cytometric features of canine peripheral T-cell lymphoma, not otherwise specified (PTCL, NOS). Architecturally, the neoplastic infiltrate is diffuse (**A**) and the infiltrating cells are heterogeneous, with cell size varying from small to large with pleomorphic, oval to cleaved nuclei (**B**). Flow cytometry of the neoplastic cells (colored green, **C**–**E**) in reported cases of PTCL, NOS reveals cells that are large by light scatter (**C**), CD5^+^, CD4^+^ (**D**), and demonstrate decreased expression of MHC Class II (**E**). These findings contrast to the T-cells within a normal canine lymph node (green, **F**–**H**), which are small by light scatter (**F**), CD5+, split between CD4^+^ and CD4^−^ (**G**), and demonstrate higher levels of MHC Class II expression (**H**).

The diagnosis of PTCL, NOS in either species, because it lacks any single specific morphologic, genetic, or immunophenotypic signature, is considered to be a process of exclusion. It is noteworthy that this pathologic heterogeneity has resulted in poor (*i.e.*, 75%) diagnostic agreement amongst human hematopathologists in the diagnosis of PTCL, NOS [57]. Similar studies have not been conducted in veterinary hematopathology. Histologically, both human and canine PTCL, NOS are usually diffuse diseases although rare follicular variants of hPTCL, NOS are recognized (Figure 4) [58]. The cytomorphology of the neoplastic cells in both species is highly variable and can include varying proportions, either monomorphic or polymorphic, of small, medium, or large cells. Reflective of this, a previous canine-adapted version of the Kiel classification scheme divided PTCL, NOS into 6, morphologically-unique subtypes, namely pleomorphic small-cell, small clear-cell, pleomorphic mixed, pleomorphic large-cell, immunoblastic, and plasmacytoid lymphomas [11]. However in both species, most cases consist of medium-to-large sized cells with irregular nuclei, a variable chromatin pattern, prominent nucleoli, and variable mitotic activity [15,39,40,59].

There is no consistent immunophenotypic signature for hPTCL, NOS and specific immunophenotypes do not consistently correspond to a clinically-meaningful entity [60]. However, neoplastic cells in both human and canine PTCL, NOS are typically positive for mature T-cell markers, including CD3 and CD5 [39,40]. Phenotypic aberrancy in hPTCL, NOS, as evident by loss of either CD5 and CD7 or CD4/CD8 dual positivity or negativity are also common [59,60,61]. Nodal cases of hPTCL, NOS are most commonly CD4+/CD8− [56]. Additional commonly expressed markers in hPTCL, NOS include TIA-1 (T-cell intracellular antigen-1), CXCL13, PD-1, CD52, and CD30 [62,63,64], and many of these markers help to support a helper *vs.* cytotoxic differentiation. Although the extent of the immunophenotype of cPTCL, NOS has not been described, like its human counterpart, the nodal variant is most commonly reported as a CD45+, CD3+, CD4+, CD8−, MHC II low, CD21 negative disease, with frequent loss of CD5 expression [39,40,41].

### 4.2. T-Zone Lymphoma

T-zone lymphoma (TZL) is a variant of both human and canine T-cell lymphoma characterized by a unique histologic and cytologic appearance. Although no publications directly address its incidence, TZL appears to account for less than 2% of human PTCLs [65]. Although the true incidence of cTZL has not been defined it appears to be much more common in veterinary medicine, accounting for 16%–62% of all canine indolent lymphomas. In the current WHO guidelines, human TZL (hTZL) is classified as a *morphologic variant* of PTCL-NOS rather than a *distinct subtype* of NHL [56]. In contrast, due to its clinical, histologic, and immunophenotypic novelty, canine TZL (cTZL) is widely accepted to be distinct subtype of canine lymphoma.

**Figure 5:**
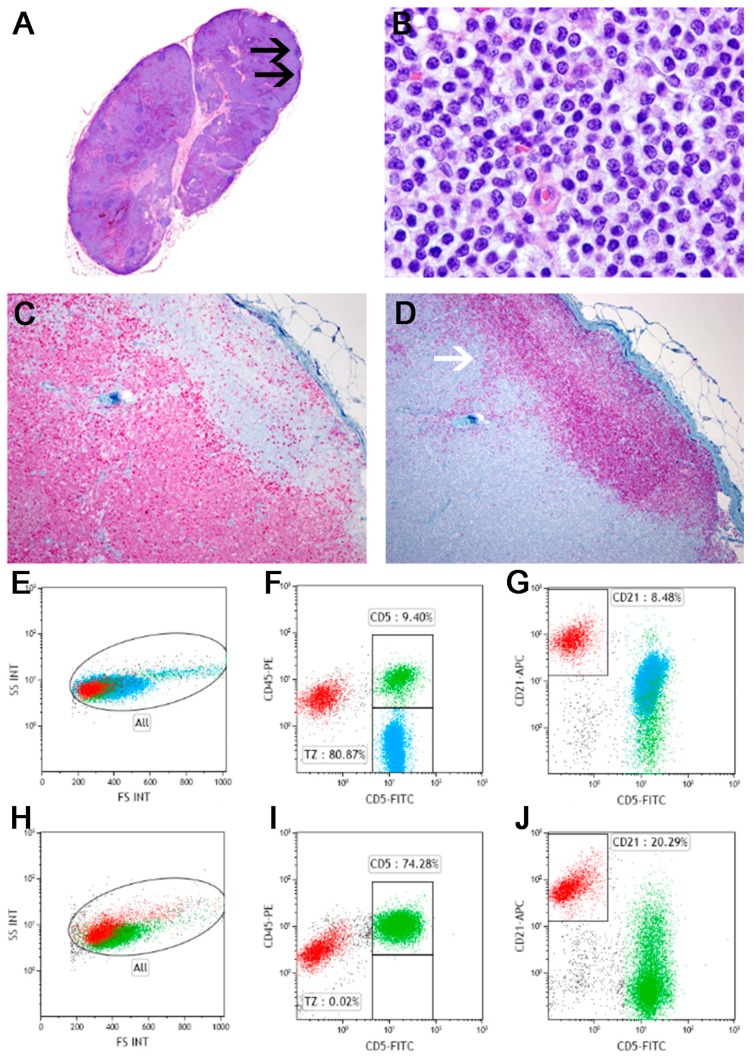
The histologic, immunohistochemical, and flow cytometric appearance of canine T-zone lymphoma (TZL). Affected nodal tissue is characterized by expansion and effacement of the T-cell areas of the cortex and medulla with resulting capsular displacement and compression of residual follicles (black arrows, **A**). The neoplastic cells are intermediately-sized and contain a moderate amount of pale blue cytoplasm and ovoid nuclei with sharp, shallow indentations, finely granular chromatin, and inapparent nucleoli (**B**). Anti-CD3 (red, **C**) and anti-Pax5 (red, **D**) immunohistochemistry demonstrates the T-cell phenotype of the neoplastic cells and further illustrates the compressed, B-cell rich residual follicles (white arrow, **D**). Flow cytometrically, in contrast to a normal canine peripheral lymph node (**H**–**J**), TZL (**E**–**G**) is characterized by the presence of an intermediate-sized population of CD5+ cells (colored blue) which are phenotypically aberrant owing to their lack of CD45 expression (**F** *vs.* **I**) and increased CD21 expression (**G** *vs.* **J**).

Although the diagnosis of TZL in both species necessitates tissue evaluation and recognition of its novel histologic and cytologic features, Seelig *et al.* report that its unique immunophenotypic features may allow the diagnosis of cTZL to be made by flow cytometry alone [66]. According to the original Kiel classification scheme, hTZL is non-leukemic type of lymphoma containing all components of the T-regions of lymphoid tissue and can be considered as the diametric counterpart to follicular lymphoma [67]. Architecturally, in both canine and human, TZL is a non-effacing disease in which there is retention of B-cell follicles, including germinal centers, in the face of dramatic inter-follicular expansion owing to proliferating neoplastic T-cells (Figure 5) [67,68]. Expansion of the interfollicular space by the neoplastic population results in displacement and compression of the residual follicles against a thinned perinodal capsule [66]. Owing to its non-effacing nature, TZL can be classified as a nodular disease. Cytologically, the neoplastic cells in similar in both canine and human TZL. In both species, they are small to medium-sized with a moderate amount of lightly stained cytoplasm and oval to elliptic nuclei with sharp, shallow indentations, finely granular chromatin, and inapparent nucleoli [67,69]. In both species, small numbers of large cells may be noted. In cTZL, mitotic figures are generally rare, but the human form they are described as either uncommon or very frequent [67,68]. In cases in which mitotic figures are evident, their significance is uncertain. Anecdotally, the neoplastic cells in cTZL are often described as having cytoplasmic tails, which are described on cytology samples, as either “hand mirrors” or “uropods” [70].

In contrast to cTZL, human patients with TZL are described as seldom showing peripheral blood involvement [67]. In the dog, peripheral blood lymphocytosis is described in 48%–64% of cases and neoplastic cells can be detected in the peripheral blood in 100% of cases in which peripheral blood was available for flow cytometric analysis, including many cases in which the absolute lymphocyte count was normal [16,66,69]. Bone marrow involvement in both hTZL and cTZL appears uncommon and peripheral blood cytopenias are very uncommon [69,71]. Limited hTZL-specific immunophenotypic information is available, but the tumor cells are described as CD2+, CD3+, CD5+, and CD4+ [72]. To date, no studies of hTZL using modern immunophenotyping techniques or reagents have been reported. The immunophenotype of cTZL is typical of a mature T-cell neoplasm through its expression of both CD3+ and CD5+ and the lack of for B-cell antigens CD20, Pax5, and CD79a. However, TZL is unique among canine lymphoma through its CD45− and CD21+ phenotype [66,69]. The expression of CD4 and CD8 is variable as single positive, dual positive, and dual negative variants have been described [66,69,73].

## 5. B-Cell Neoplasms

B-cell lymphomas arise during different steps of B-lymphocyte development and represent their malignant counterpart and, as such, pre-germinal and post-germinal center lymphomas can be identified [74]. In both man and dog, the B-cell phenotype is the most common form of lymphoma, representing 85%–90% and 62%–75% of all human and canine lymphomas, respectively [11,13,17].

### 5.1. Diffuse Large B-Cell Lymphoma

In both dog and man, diffuse large B-cell lymphoma (DLBCL) is the most common mature lymphoid malignancy. In humans, DLBCL represents the most common form of adult NHL and the most common form of BCL, representing 30%–40% of all adult NHL and 37% of BCL [17,31]. Similarly, DLBCL is the most common form of lymphoma in the dog, although the overall incidence is higher, representing 47%–56% of all canine lymphomas and 73% of canine BCL [11,13]. Although the neoplastic cells share generally similar morphologic features between the two species, human DLBCL (hDLBCL) can be subdivided according to genetic, immunophenotypic, and molecular studies into four subgroups: (1) DLBCL—not otherwise specified (NOS); (2) DLBCL with a predominant extranodal location; (3) Large cell lymphomas of terminally differentiated B-cells, and (4) B-cell neoplasms with intermediate features (aka borderline cases) [17]. At present, although the morphologic and immunophenotypic heterogeneity of cDLBCL has not been reported, there is evidence suggesting genetic variation is likely to exist in cDLBCL [75,76] and that such genetic variation correlates with biologic behavior [77].

In both species, DLBCL most commonly occurs as a *de novo* disease, although transformation from a less aggressive BCL, including MZL and FL (*i.e.*, Richter syndrome/Richter transfromation) is well-characterized in hDLBCL. [78]. Although similar events have been described in the dog, further work is needed to define its pathologic scope and disease-specific frequency [79]. Work by Frantz *et al*. by demonstrating molecular overlap between MZL and DLBCL, in concert with the morphologic overlap between these two entities, suggests that an unknown percentage of cases of cDLBCL might represent transformed cMZL [77]. Clinically, both canine and human DLBCL patients present with rapidly progressive, generally painless nodal or extranodal disease with clinical signs reflecting tumor burden and location [80]. Hematologically, approximately 10%–25% of hDLBCL cases present with peripheral blood involvement at the time of diagnosis diagnosis (more likely to be detected with sensitive techniques such as flow cytometry) and, although the frequency of blood involvement in dogs is less certain, one study found that in 50% of 14 cases, neoplastic B-cells could be found in peripheral blood by flow cytometry [81,82,83].

**Figure 6:**
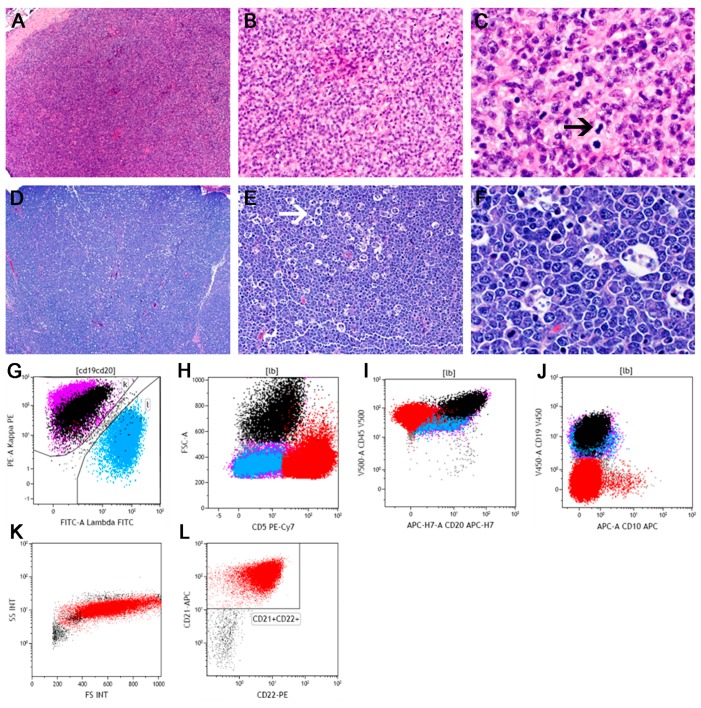
The histologic and flow cytometric features of human and canine diffuse large B-cell lymphoma (DLBCL). In both species, DLBCL is a diffuse disease (**A**–**C**, human and **D**–**F**, canine) and often demonstrates a starry-sky appearance due to phagocytic histiocytes (white arrow, **E**). Neoplastic cells are large with varying amounts of cytoplasm and vesicular nuclei with prominent nucleoli. The mitotic rate is often high (black arrow, **F**). Flow cytometrically, the neoplastic cells (colored black) in hDLBCL are light chain restricted (**G**), large by forward light scatter, CD5− (**H**), CD45+, CD20+ (**I**), CD19+, and CD10− (**J**). Similarly, the neoplastic cells in cDLBCL (colored red) are large by forward scatter (**K**) and are CD21+ and CD22+ (**L**).

The definitive diagnosis of both human and canine DLBCL requires histopathological examination of affected tissues [27,80]. Architecturally both hDLBCL and cDLBCL are diffuse diseases with coagulative necrosis and extension into the surrounding tissue (Figure 6) [15,84]. At low magnification, a “starry-sky” appearance resulting from the scattered representation of histiocytes with phagocytosed cellular debris is commonly observed in many cases [18,84]. The presence of remnant follicular structures may be apparent, including germinal centers and mantle zone cells. Cytomorphologically, a defining feature of DLBCL in both species is the size of the neoplastic cell. In hDLBCL, the cells are described as intermediate to large with nuclei at least twice the size of a small lymphocyte and usually larger than a tissue macrophage or endothelial cell nucleus [78]. In cDLBCL, the cells are similarly large. In both species, the number of mitotic figures is typically quite high and the amount of reactive T-lymphocytes and/or histiocytes is variable [78].

According to their nuclear morphology, neoplastic cells in both human and canine DLBCL are commonly characterized as resembling centroblasts (CB), immunoblasts (IB), or a form intermediate to the two. Centroblasts are medium to large-sized cells with round to oval nuclei with vesicular chromatin, multiple small nucleoli opposed to the nuclear membrane, and a thin rim of amphophilic cytoplasm. Immunoblasts are similarly sized with round to oval vesicular nuclei, a single, large centrally-located nucleolus, and a broad rim of basophilic cytoplasm. Based upon the percentage of either cell type, DLBCL can be cytologically subclassified as either immunoblastic (*i.e.*, > 90% immunoblasts) or centroblastic (*i.e.*, >90% centroblasts) [18,80]. In hDLBCL, the clinical relevance of morphologic subclassification is inconclusive as some studies have reported a worse outcome for immunoblastic hDLBCL, whereas others have not [85,86]. To date, no studies have established any clinical significance to the stratification of canine lymphoma into IB or CB subtypes. There is no characteristic immunophenotype for either human or canine DLBCL as both express pan-B markers, including CD79a, Pax5, and CD20, and CD21 [27,84].

According to gene expression profiling (GEP), hDLBCL can be further segregated into three molecular subtypes, namely germinal center B-cell-like (GCB-DLBCL), activated B-cell-like (ABC-DLBCL), and primary mediastinal B-cell lymphoma (PMBL). Each of these subtypes has notable biological and therapeutic differences, illustrating the prognostic significance of such an approach [87,88,89]. Given the practical limitations of GEP as a primary diagnostic modality, IHC has been proposed as a surrogate. The most commonly used algorithm (Hans) segregates DLBCL into GCB-type and non-GCB types according to their expression of CD10, BCL6, and IRF4/MUM-1 [90]. Attempts to stratify canine DLBCL into GCB and ABC-like categories using gene expression data have been limited, but have shown that GEP can organize canine BCL into multiple subtypes with prognostic implications [75,77]. However, the limited attempts to apply the Hans algorithm to canine large cell lymphomas have not been successful [75].

### 5.2. Marginal Zone Lymphoma

The marginal zone lymphomas (MZLs) are a group of similarly named, but biologically unique forms of mature B-cell neoplasia that originate from post-follicular memory lymphocytes of the marginal zone, which is a unique antigen filtration region of the spleen and lymph node [91]. In both humans and dogs, three variants of MZL are described, namely: (1) nodal marginal zone lymphoma (NMZL), (2) splenic marginal zone lymphoma (SMZL) and (3) mucosal associated lymphoid tissue lymphoma (MALT lymphoma) [17]. In all subtypes of both hMZL and cMZL, the neoplastic cells are characterized by many common morphologic, histologic, and immunophenotypic characteristics [91]. Despite this, each has very unique diagnostic features as well as recurrent cytogenetics/translocations.

#### 5.2.1. Splenic Marginal Zone Lymphoma

Splenic marginal zone lymphoma (SMZL) is an indolent, small-cell B-cell lymphoma characterized by a unique pattern of splenic infiltration and widespread bone marrow involvement (with characteristic intrasinusoidal distribution). It was formally included in the WHO classification system in 2001 and, despite its MZL nomenclature, it is a distinct from either NMZL and MALT lymphoma [92]. Splenic MZL is uncommon form of human B-cell lymphoma that accounts for 0.6% of cases of NHL and 20% of hMZLs [36]. It is a similarly uncommon in the dog accounting for 0.2%–1.0% of canine lymphoma and 2%–16% of cMZLs [11,13].

Clinically, hSMZL is characterized by generalized splenomegaly with concurrent peripheral blood and bone marrow involvement with minimal to absent lymphadenopathy [32,34,36,93]. In contrast, dogs with SMZL commonly present with either a solitary splenic mass in (79%) or multiple splenic nodules (24%) whereas generalized splenomegaly is uncommon [33,45,68]. Moreover, the frequency of peripheral blood involvement in cSMZL as documented by a peripheral blood lymphocytosis appears to be much lower (2.9% *vs.* 90%) than its human counterpart [16,33]. In contrast, hemoabdomen as a result of spontaneous splenic rupture is far more common in the cSMZL (22% of cSMZL) as compared to hSMZL, where it is very rarely described [33,44,45,68,94].

The diagnosis of hSMZL requires the identification of a mature B-cell neoplasm with a unique pattern of tissue involvement, cytology, and characteristic cytogenetic abnormalities. Architecturally, the tumor infiltrate is nodular and consists of small lymphocytes that surround and replace the splenic white pulp germinal centers, efface the follicle mantle zone, and merge with a peripheral zone of larger cells [35]. The architecture of canine SMZL is similar to its human counterpart and is characterized by a multi-nodular infiltrate in which the number and size of white pulp follicles, which often encircle fading remnants of germinal centers, is increased and begin to coalesce [11,15,45,68]. Cytomorphologically, hSMZL appears to be slightly distinct from cSMZL. In the former, infiltrated follicles maintain a biphasic appearance with a centrally located small lymphocyte population with round nuclei and scant cytoplasm, surrounded by a cuff of larger cells [35]. These larger cells are slightly larger than the small cells with slightly irregular nuclear contours and moderate to abundant pale cytoplasm [37]. While morphologically distinct, both the central core of small lymphocytes and the outer zone of larger cells have been shown to be part of the neoplastic clone [92]. In contrast, the neoplastic cells in cSMZL are reported to be more monomorphic [95]. They are medium-sized and contain an intermediate amount of lightly-stained pink cytoplasm. Nuclei are vesicular with peripheralized chromatin and a prominent single central nucleolus [15]. In both species, mitotic figures are rare [95] and neoplastic areas may be surrounded by areas of hemorrhage or EMH [45]. Immunophenotypically, neoplastic cells in hSMZL are CD20+, IgD+, BCL2+, CD3−, CD5−, CD10−, and BCL6− [51]. In the dog, neoplastic SMZL cells are described as CD79a and CD20 positive and CD3 negative [95,96].

#### 5.2.2. Nodal Marginal Zone Lymphoma

Nodal marginal zone lymphoma (NMZL), like the other forms of MZL, is a primary B-cell neoplasm originating from marginal zone cells. Morphologically, NMZL is composed of tumor cells similar to those encountered in either extranodal or splenic subtypes of MZL, however the disease burden is isolated to lymph nodes. As such, diagnosing NMZL requires knowledge of disease distribution [97]. Human NMZL (hNMZL) is uncommon, accounting for less than 2% of all NHLs and approximately 10% of all MZLs [91]. In contrast, NMZL is a more common disease in dogs, accounting for 6%–10% of all forms of canine lymphoma and 75%–89% of all cMZLs [11,12,13]. Clinically, hNMZL is characterized by peripheral lymphadenopathy without evidence of either extranodal or splenic sites. In hNMZL, bone marrow involvement is apparent in 30%–45% of cases, although peripheral blood involvement is rather rare (approximately 10%) and cytopenias are infrequent. Clinically, canine NMZL (cNMZL) presents as single or generalized lymph node enlargement and no studies have evaluated bone marrow or peripheral blood involvement [13]. The diagnosis of both NMZL can be challenging in both species as it lacks a specific clinical, morphological, or immunophenotypic signature.

**Figure 7:**
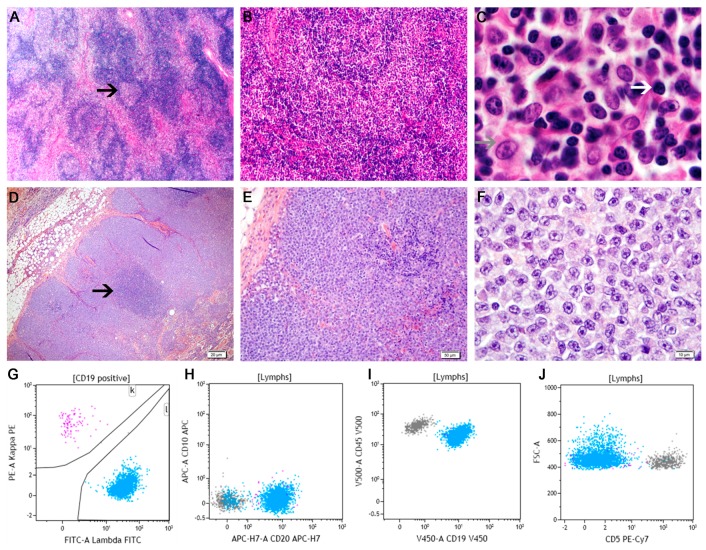
This histologic and flow cytometric appearance of human and canine nodal marginal zone lymphoma (**A**–**C**, human and **D**–**F**, canine). At low magnification both species demonstrate a nodular architectural pattern characterized by expansion of germinal centers and perifollicular areas (**A, D**) by a population of intermediate-sized neoplastic cells. In both, residual follicular structures are common (black arrow, **A** and **D**). At higher magnification, the neoplastic infiltrate in hMZL consists of transformed intermediate (white arrow, **C**) and large cells (gray arrow, **C**). In contrast, neoplastic cells in cMZL are predominately intermediate sized with abundant pale cytoplasm and irregular nuclei containing peripheralized chromatin and a single central nucleolus (**F**). Flow cytometrically, the neoplastic cells (colored blue, **G**–**J**) in hMZL are lambda light chain restricted, CD20+, CD45+, CD19+, CD5−, and CD10−.

Further challenging the diagnosis of NMZL is its heterogeneous histology, which may be more dramatic in the human variant of the disease. Architecturally, hNMZL can present with a number of patterns, including nodular, perifollicular, interfollicular, diffuse, and inverse follicular variants (Figure 7) [91,98]. In each, there may be sporadic retention of normal nodal architecture, including residual germinal centers that may or may not be colonized by tumor cells [91]. In contrast, cNMZL appears to have a more uniform histology. Architecturally, cNMZL is reported to be nodular resulting from the expansion of perifollicular marginal zone lymphocytes surrounding fading remnants of germinal centers (Figure 7) [15]. Like its architecture, the cytologic appearance of hNMZL is also heterogeneous. Although the neoplastic cells in hNMZL are generally medium in size, they lack the monotony seen in seen in other forms of lymphoma. Most cases contain two types of cells, namely: (1) a dominant, small to medium-sized population containing pale cytoplasm and nuclear irregularities (*i.e.*, centrocyte-like); and (2) a minority large cell population with oval to reniform nuclei and abundant pale cytoplasm (*i.e.*, monocytoid). Canine NMZL is also a mixed cell disease, although the dominanT-cell type is centrocyte-like in its morphology, namely intermediate sized with abundant pale cytoplasm and irregular nuclei containing peripheralized chromatin and a single central nucleolus (Figure 7) [68].

While hNMZL is considered to an indolent form of B-cell lymphoma, transformation to large-cell disease (*i.e.*, DLBCL) is reported to occur in 7%–31% of patients [97,99,100,101,102,103]. However it is important to note that the histologic criteria for hNMZL transformation are not well-defined. Proposed hNMZL transformation criteria include blast percentage (transformation defined as the large, centroblast or immunoblast percentage exceeding 20% or 50% of all tumor cells) or the identification of “sheets” of such cells. The presence of sheets of large cells is the most agreed upon marker of transformation as no difference in survival can be detected using a 20% cutoff criteria, whereas but sheets of large cells may be associated a worse clinical outcome [97,104]. Similarly, transformation of cNMZL to cDLBCL is thought to occur [18] and, correlate with this, gene expression profiling has revealed that the two diseases cannot be distinguished [77]. Although as in human hematopathology, there are no defining histologic criteria for this process and differentiating cNMZL from cDLBCL can be very challenging. Features reported to be suggestive of cNMZL include: (1) the identification of nodular foci of proliferation; and (2) intermediate (rather than large) nuclear size [13]. One author has proposed grading cNMZL according to its stage of development as “early, mid or late” based upon the area of tissue involved, the degree of coalescence of neoplastic proliferation, and the number of cells in mitosis [68]. It is unclear if this grading offers any prognostic or therapeutic implications. In summary, the importance of large cells in both canine and human NMZL continues to be debated by pathologists and whether or when these cells reflect a either a morphologic variant of MZL or true transformation to DLBCL.

The immunophenotype of hNMZL is identical to other hMZL variants, including expression of mature B-cell antigens (CD19, CD20, CD22, CD79a) and an absence of germinal center antigens (CD10, bcl-6, cyclin D1) [91]. The neoplastic cells in cNMZL demonstrate a mature B-cell phenotype as reflected by CD79a+, CD20+, and CD3−, and nearly all cases demonstrate clonal IgH rearrangements with a small fraction having both clonal IgH and TCRg rearrangements [16,68].

### 5.3. Mantle Cell Lymphoma

In both humans and dogs, mantle cell lymphoma (MCL) is an uncommon form of B-cell lymphoma, representing 3%–6% and 0.7%–1.8% of NHLs in the two species, respectively [11,13,105,106]. However, this is largely where the similarities end. Clinically, human MCL (hMCL) is an intermediate-grade disease characterized by generalized lymphadenopathy and frequent extranodal involvement, including spleen, bone marrow, liver, and GI tract infiltration [107]. In humans, peripheral blood involvement can be identified in up to 70% of patients on routine examination and this number increases to nearly 100% with flow cytometry [108,109,110]. Bone marrow involvement is reported in 50%–91% of patients and cytopenias, most notably anemia and thrombocytopenia, are seen in up to 40% of patients [108,111,112]. In contrast, canine MCL (cMCL) primarily affects the spleen (80% of the reported cases are of splenic origin) with only occasional visceral node involvement [11,13,16,68]. The vast majority of cases lack detectable peripheral node infiltration and peripheral blood or bone marrow involvement appears to be very uncommon. Biologically, cMCL is reported to be an indolent disease and a series of nine dogs with splenic MC had a 1 year survival rate of 88.9% and an overall survival period of 502 days [113].

**Figure 8:**
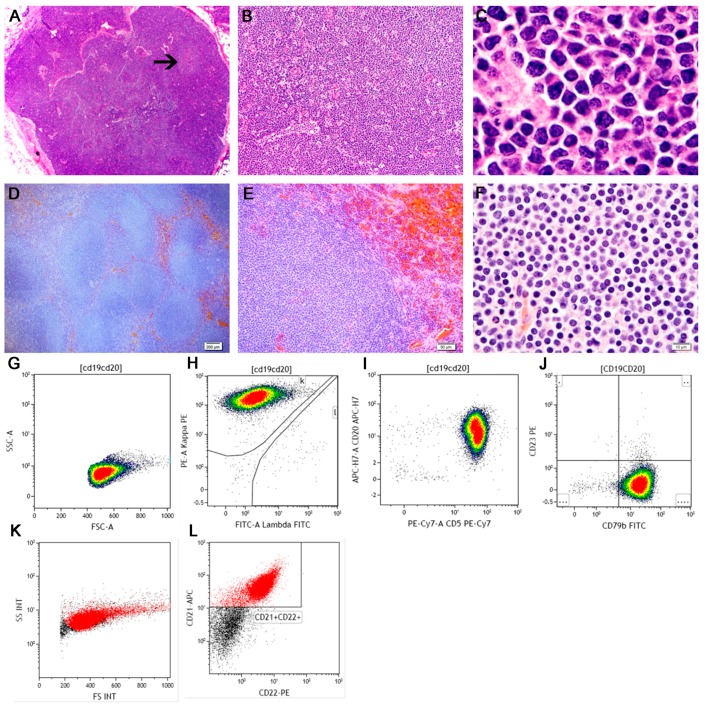
The histologic and flow cytometric features of human and canine mantle cell lymphoma (**A**–**C**, human and **D**–**F**, canine). Architecturally, hMCL is a heterogeneous disease, although the nodular (mantle zone) pattern is presented (**A**, **B**). The tumor infiltrate expands the mantle cell cuff and surrounds a reactive germinal center (black arrow, **A**). The neoplastic cells are small to intermediately sized, contain scant cytoplasm and demonstrate indented nuclear contours with evenly dispersed chromatin (**C**). Architecturally, cMCL is characterized by discrete to coalescing nodules of a monotonous population of neoplastic cells (**D**, **E**). Morphologically, these cells are small to intermediate in size with round nuclei, condensed chromatin, and inapparent nucleoli (**F**). By flow cytometry, the cells are intermediate-sized by forward scatter (**G**), light chain restricted (**H**), CD20+, CD5+ (**I**), CD79b+, and CD23− (**J**). The neoplastic cells in cMCL (colored red) are small to intermediate in size (**K**) and are CD21+ and CD22+ (**L**).

The diagnosis of hMCL is based upon the identification of a mature B-cell neoplasm composed of small to intermediate-sized cells with a unique immunophenotypic and genetic profile [108]. Architecturally, hMCL is characterized by three possible growth patterns, two of which are nodular (the mantle zone and nodular forms) and one of which is diffuse (the diffuse form) (Figure 8) [108]. The mantle zone pattern results in expansion of the follicle mantle area by tumor cells surrounding a reactive germinal center whereas the nodular pattern consists of nodules of tumor cells that lack a remnant germinal center [108]. The diffuse form of the disease is characterized by sheets of neoplastic cells in which rare residual germinal centers may be seen. The architectural heterogeneity of hMCL has not been described in cMCL and, to date, it is described primarily as a nodular disease although diffuse areas may be seen in later stages of the disease [18]. The nodules of neoplastic cells in cMCL, which may coalesce, consist of a monotonous population of cells that are often centered around end arterioles and/or a faded, hypocellular germinal center (Figure 8) [18]. The architecture of cMCL has been described as the histologic inverse of cMZL [18].

Morphologically, hMCL is similarly heterogeneous and four morphologies are described, namely classic (common or typical), small, blastoid, or pleomorphic variants [114]. In the classical form, the cells are monomorphic, small to medium in size, contain scant cytoplasm and demonstrate indented nuclear contours, evenly dispersed chromatin, scan cytoplasm, inapparent nucleoli, and few mitoses [17]. Rare large cells may be seen. The small cell form is dominated by small lymphocytes with round nuclei; the blastoid form is composed of medium-sized tumor cells with round nuclei, finely dispersed chromatin, and numerous mitoses, whereas the pleomorphic variant is comprised of large cells with irregular nuclear morphologies [108]. The classic and small-cell forms of the disease typically have a low mitotic index and cases with increased mitotic activity are associated with a worse prognosis [108]. In contrast to the other morphologic manifestations, the blastoid and pleomorphic variant have poor prognoses. Like its architecture, the cytomorphology of cMCL is more uniform than its human counterpart. The neoplastic cells are small to intermediate in size and contain a scant amount of cytoplasm and round to rarely indented nuclei containing dense chromatin, and inconspicuous nucleoli. The mitotic rate is very low to high [11,68].

In light of this histologic heterogeneity, immunophenotyping is mandatory for the diagnosis of hMCL [107]. Phenotypically, hMCL is a mature B-cell neoplasm characterized by positivity for the B-cell markers CD19, CD20, CD22, CD79a, CD79b, with surface Ig expression and lambda light-chain restriction in approximately 2/3rds of cases [107]. Unique among other forms of B-cell neoplasia is the near-ubiquitous expression of the T-cell associated antigen CD5 and the expression of cyclin D1 [107,108]. Moreover, most cases of hMCL are negative for CD10 and Bcl6 (which aids in excluding follicular lymphoma, DLBCL, and precursor disease). Immunophenotypically, cMCL is a mature B-cell neoplasm characterized by CD79a and CD20 expression [12,16].

### 5.4. Follicular Lymphoma

Follicular lymphoma (FL) is a mature B-cell neoplasm with similar pathologic features between dog and man, but very different incidences. Whereas FL is the second most common form of human lymphoma, representing 20% of NHL, canine FL (cFL) is quite rare and reported to represent less than 1% of canine lymphomas [11,15,31]. Clinically, most human patients with FL have generalized lymph node involvement, including peripheral, mediastinal, and retroperitoneal nodes [115,116]. Bone marrow involvement is present in a majority of patients at staging [116]. Given its rarity, the clinical features of cFL are scarcely reported, but as in human FL (hFL), multinodal disease including both peripheral and visceral nodes, appears common [68] . In both species, the magnitude of lymph node enlargement is mild to moderate and generally less than other forms of NHL [18,117].

**Figure 9:**
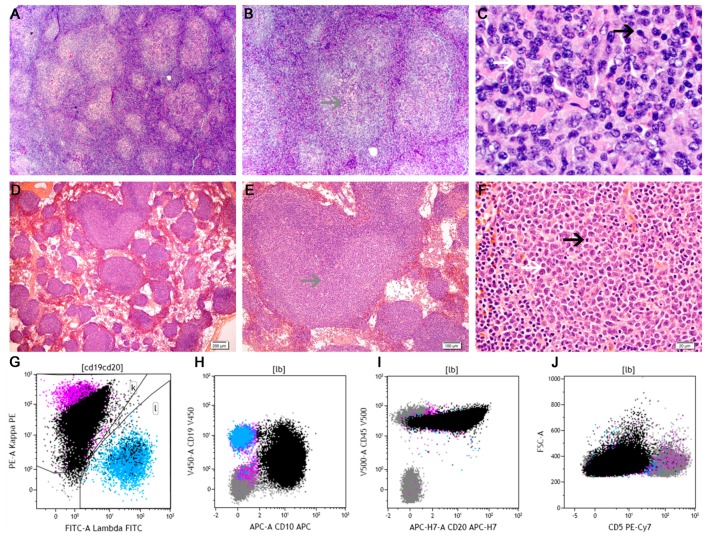
The histologic features of human and canine follicular lymphoma (**A**–**C**, human and **D**–**F**, canine). Architecturally, in both species, the disease is nodular (**A, C**) and the neoplastic follicles lack germinal center polarization (gray arrow, **B** and **E**). Morphologically, the tumor infiltrate in both species is bimorphic, composed of predominantly smaller neoplastic centrocytes (black arrow, **C** and **F**) and rare admixed larger neoplastic centroblasts (white arrow, **C** and **F**). By flow cytometry, the neoplastic cells in human FL (colored black) are light chain restricted (**G**), CD10+, variably CD19+ (**H**), CD20+, CD45+ (**I**), but CD5−, and are intermediate size by forward light scatter (**J**).

In both species, FL is best diagnosed using excisional lymph node biopsy and FNA samples are not considered adequate [18,68,116]. Both hFL and cFL are nodular diseases characterized by complete effacement of the nodal architecture by neoplastic follicles (Figure 9). Owing to its unique architecture, distinguishing the neoplastic follicles in FL from the benign follicles in reactive hyperplasia can be challenging. However, neoplastic follicles lack two architectural features seen in their benign counterpart, namely: germinal center polarization and a well-defined and continuous mantle zone cuff. Germinal center polarization is a histologic hallmark of antigen-induced lymphoid hyperplasia and reflects the proliferation of distinct mature B-cell populations. Histologically, germinal center polarization manifests as segregation of the germinal center into two zones, namely an apical light zone, composed of small-to-medium sized cells that contain a relatively abundant amount of pale-staining cytoplasm (*i.e.*, centrocytes) and a deeper dark zone containing large cells that contain a narrow rim of dark-staining cytoplasm (*i.e.*, centroblasts).

Cytologically, although the neoplastic follicles in both human and canine FL lack polarization, they are composed of cells that are morphologically similar to those in normal germinal centers. Neoplastic *centrocytes* are small cells with clear cytoplasm, an irregular nuclear outline, pale and evenly dispersed chromatin, and inconspicuous or small nucleoli. Neoplastic *centroblasts* are large cells with a moderate amount of dark basophilic cytoplasm, round to oval to indented, vesicular nuclei with peripherally-dispersed chromatin and 1–3, peripherally-positioned nucleoli [11,68,118]. In both species, centrocytes are usually more common than centroblasts in the neoplastic follicles and mitotic activity is generally low [116]. In addition to follicle architecture, FL can be differentiated from lymphoid hyperplasia through the detection of intravascular invasion or a more numerous and more monotonous centrocyte population, the absence of an appreciable plasma cell population or starry-sky macrophages, irregular-shaped follicles with attenuated mantle zones, follicles arranged “back-to-back,” and extension of cells beyond the capsule into perinodal tissues [116,118].

Human FL can be further graded by counting the number of centroblasts in 10–20, randomly-selected 400× microscopic high-power fields (hpf) into grade 1 (1–5 centroblasts), grade 2 (6–15 centroblasts), or grade 3 (>15 centroblasts) forms of the disease [17]. Grade 3 FL can be further split into “A” and “B” forms of the disease, with grade 3A demonstrating more than 15 centroblasts per hpf but with a persistent centrocyte population and persistent of a follicular dendritic cell meshwork whereas grade 3B forms have solid sheets of centroblasts and lack a follicular dendritic cell meshwork [116]. While grade generally correlates with disease aggressiveness, there is disagreement regarding the interobserver reproducibility and clinical significance of this system [117,119]. Accordingly, in light of their shared clinical behavior, grades 1 and 2 hFL have recently been combined into a single “low grade” category, termed WHO grade 1–2 [116]. To date, likely owing to its rarity, a histologic grading scheme for cFL has not been systematically evaluated. Immunophenotypically, neoplastic cells in hFL express typical B-cell antigens (CD19, CD20, CD22, CD79a, and PAX5) and most cases express the germinal center-associated protein CD10 and BCL6 [116,118,120]. Similarly, cFL has been shown to express mature B-cell markers (CD79a) while demonstrating a lack of staining with mature T-cell markers (*i.e.*, CD3) [11,12,16,68].

## 6. Conclusions

In light of their complementary biology, including overlapping incidence, genetic, and clinical features, there is tremendous interest in utilizing the dog as an animal model for human NHL. However, implicit in this approach is a need to determine the degree to which canine and human lymphomas are diagnostically similar. While the high reproducibility, and subsequent increasing acceptance, of canine-adapted WHO guidelines may be a watershed moment in veterinary hematopathology, subsequent studies are needed to further refine these guidelines, confirm their clinical and diagnostic utility, and identify and validate novel reagents and techniques.

## Figures and Tables

**Table 1:** Canine and human non-Hodgkin lymphomas according to the 2008 WHO guidelines.

*B-Cell Neoplasms*	*T-Cell Neoplasms*
**B-lymphoblastic leukemia/lymphoma**	**T-lymphoblastic leukemia/lymphoma**
**Chronic lymphocytic leukemia/small lymphocytic lymphoma**	T-cell prolymphocytic leukemia
B-cell prolymphocytic leukemia	**T-cell large granular lymphocytic leukemia**
**Splenic marginal zone lymphoma**	Chronic lymphoproliferative disorder of NK cells
Hairy cell leukemia	Aggressive NK-cell leukemia
Splenic lymphoma/leukemia, unclassifiable	EBV-positive T-cell disease of childhood
**Lymphoplasmacytic lymphoma**	Hydroa vacciniforme-like lymphoma
Heavy chain diseases	Adult T-cell leukemia/lymphoma
**Plasma cell myeloma**	Extranodal NK/T-cell lymphoma, nasal type
**Solitary plasmacytoma of bone**	**Enteropathy-associated T-cell lymphoma** *
**Extraosseous plasmacytoma**	**Hepatosplenic T-cell lymphoma**
MALT lymphoma	Subcutaneous panniculitis-like T-cell lymphoma
**Nodal marginal zone lymphoma**	**Mycosis fungoides**
**Follicular lymphoma**	**Sézary syndrome**
Primary cutaneous follicle centre lymphoma	Cutaneous CD30^+^ T-cell disorders
**Mantle cell lymphoma**	**Peripheral T-cell lymphoma, NOS**
**Diffuse large B-cell lymphoma**	T-Zone lymphoma **
**Lymphomatoid granulomatosis**	Angioimmunoblastic T-cell lymphoma
Primary mediastinal large B-cell lymphoma	Anaplastic large cell lymphoma, ALK-positive
Intravascular large B-cell lymphoma	Anaplastic large cell lymphoma, ALK-negative
ALK-positive large B-cell lymphoma	
Plasmablastic lymphoma	
Large B-cell lymphoma in Castleman disease	
Primary effusion lymphoma	
**Burkitt lymphoma**	
B-cell lymphoma, unclassifiable	

Non-bolded entities represent malignancies in humans only whereas bolded entities reflect malignancies reported in both humans and canines. * A neoplasm with an EATL-like morphology has been described in dogs, but an association with celiac disease has not been documented. ** In humans, TZL is considered a morphologic variant of PTCL, NOS.

**Table 2:** Comparative histologic and immunophenotypic characteristics of common forms of Canine and human non-Hodgkin lymphoma.

Disease	Canine		Human	
	Histology	Phenotype	Histology	Phenotype
*Precursor Neoplasms*	Architecture: Diffuse	*T-cell*: CD45^+^, CD34^+/−^, CD5^+/−^, CD3^+/−^, CD4^+/−^, CD8^−^	Architecture: Diffuse	*T-cell*: CD34^+^, TdT^+^, cytoplasmic CD3^+^, CD5^+^
	Cytology: Intermediate sized cells, round nuclei, scant cytoplasm, high mitotic rate.	*B-cell*: CD45^+^, CD18^+^, CD34^+/−^, CD79a^+^, CD21^+/−^	Cytology: Small to large cells, varied cytoplasmic volume, high mitotic rate.	*B-cell*: CD34^+^, TdT^+^, CD19^+^, CD79a^+^, sIg^−^
*DLBCL*	Architecture: Diffuse	CD20^+^, CD21^+^, CD45^+^, CD79a^+^, Pax5^+^, MHC II^+^	Architecture: Diffuse	sIg^+^, CD45^+^, CD20^+^, CD22^+^, CD79a^+^, Pax5^+^, CD10^+/−^
	Cytology: Large cells with round nuclei. One (central, immunoblast) or multiple (peripheral, centroblasts) nucleoli. High mitotic rate and “starry sky” appearance.		Cytology: Large cells with round nuclei. Both immunoblasts and centroblasts. High mitotic rate and “starry sky” appearance in a subset.	
*Mantle Cell Lymphoma*	Architecture: Nodular	CD20^+^, CD21^+^, CD45^+^, CD79a^+^, MHC II^+^	Architecture: Nodular or diffuse	sIg^+^, CD19^+^, CD20^+^, CD22^+^, CD79a^+^, CD79b^+^, CD5^+^, cyclin D1^+^, CD23^−^, CD10^−^
	Cytology: Small to intermediate-sized cells; scant cytoplasm, round nuclei with dense chromatin, and inconspicuous nucleoli. Varied mitotic rate.		Cytology: Very heterogeneous; small to large cells; round to irregular nuclei; varied nucleoli and mitotic rate.	
*Splenic Marginal Zone Lymphoma*	Architecture: Nodular	CD20^+^, CD21^+^, CD45^+^, CD79a^+^, MHC II^+^	Architecture: Nodular	sIg^+^, CD20^+^, IgD^+^, BCL2^+^, BCL6^−^, CD5^−^, CD10^−^
			Cytology: Biphasic-Small cells with scant cytoplasm and round nuclei; intermediate-sized cells with abundant cytoplasm and irregular nuclei; rare mitotic figures.	
	Cytology: Intermediate-sized cells; abundant pale cytoplasm; irregular nuclei with peripheralized chromatin and, a single nucleolus; rare mitotic figures.			
*Nodal Marginal Zone Lymphoma*	Architecture: Nodular	CD20^+^, CD21^+^, CD45^+^, CD79a^+^, MHC II^+^	Architecture: Nodular to diffuse.	sIg^+^, CD19^+^, CD20^+^, CD22^+^, CD79a^+^, CD5^−^, CD10^−^, BCL6^−^, and Cyclin D1^−^
			Cytology: Intermediate-sized cells with pale cytoplasm and irregular nuclei (centrocyte-like) and large cells with abundant pale cytoplasm and irregular nuclei (monocytoid).	
	Cytology: Mixed. Mostly intermediate-sized cells with pale cytoplasm, irregular nuclei, peripheral chromatin, and one nucleolus.			
*Follicular Lymphoma*	Architecture: Nodular	CD20^+^, CD21^+^, CD45^+^, CD79a^+^, MHC II^+^	Architecture: Nodular	sIg^+^, CD45^+^, CD19^+^, CD20^+^, CD22^+^, CD79a^+^, Pax5^+^, CD10^+^, BCL6^+^, BCL2^+^
			Cytology: Similar to the dog.	
	Cytology: Mixed. Mostly small cells with clear cytoplasm, pale chromatin, and inconspicuous nucleoli (centrocytes) with fewer large cells with dark blue cytoplasm, vesicular nuclei, and 1–3 nucleoli (centroblasts).			
**T-Cell:**				
*PTCL-NOS*	Architecture: Diffuse	CD3^+^, CD79a^−^, CD21^−^, CD45^+^, CD5^+^, CD4^+/−^, CD8^+/−^	Architecture: Diffuse	CD3^+^, CD4^+^/> CD8^+/−^, CD7^+/−^, CD5^+/−^, CD2^+^, CD30^+/−^
	Cytology: Small to large (heterogeneous) with irregular nuclei, variable chromatin, prominent nucleoli, and varied mitotic activity.		Cytology: Small to large (heterogeneous) with irregular nuclei, variable chromatin, prominent nucleoli, and varied mitotic activity.	
*TZL*	Architecture: Nodular	CD45^−^, CD3^+^, CD5^+^, CD21^+^, CD4^+/−^, CD8^+/−^	*Histologic Variant of PTCL, NOS*	CD2^+^, CD3^+^, CD5^+^, CD4^+^
	Cytology: Small to intermediate-sized cells; moderate amount of pale cytoplasm; oval to elliptical nuclei with sharp, shallow indentations; nucleoli and mitotic figures are sparse.		Architecture: Nodular	
			Cytology: Small to intermediate-sized cells; moderate amount of pale cytoplasm; oval to elliptical nuclei with sharp, shallow indentations; nucleoli and mitotic figures are sparse.	
